# Interhemispheric connectivity of primary sensory cortex is associated with motor impairment after stroke

**DOI:** 10.1038/s41598-018-29751-6

**Published:** 2018-08-22

**Authors:** Ilse Frías, Faryn Starrs, Thomas Gisiger, Jeffrey Minuk, Alexander Thiel, Caroline Paquette

**Affiliations:** 1 Department of Kinesiology and Physical Education, McGill University, Montreal, Canada; 2 Centre for Interdisciplinary Research in Rehabilitation, Montreal, Canada; 3 Centre for Research on Brain, Language and Music, Montreal, Canada; 4 Department of Neurology and Neurosurgery, McGill University, Montreal, Canada; 5 Lady Davis Institute for Medical Research at SMBD Jewish General Hospital, Montreal, Canada

## Abstract

Neuroimaging-derived markers are used to model post-stroke impairment. Among these, lesion size, corticospinal-tract lesion-load (CST-LL) and resting-state functional-connectivity (rs-FC) have been correlated with impairment. It has been shown that the sensory cortex (S1) is associated with motor learning and is essential for post-stroke recovery; yet stroke-induced changes in S1 connectivity alone are yet to be investigated. We aim to determine whether interhemispheric rs-FC could be used to refine imaging models of stroke-related impairment. Subjects’ post-stroke and age-matched controls underwent rs-fMRI. Stroke-related disability was correlated with lesion size, CST-LL and interhemispheric S1 and M1 rs-FC as independent seeds. Regression analyses were performed to assess the contribution of these markers in stroke-related deficits. Post-stroke subjects showed an asymmetrical pattern of rs-FC in which affected hemisphere S1 and M1 were mostly connected with ipsi-lesional regions. Correlations between rs-FC and stroke-severity were found. Adding rs-FC of S1 to the regression model of impairment decreased the variance 31% compared to lesion size only. After a stroke, S1 interhemispheric connectivity is decreased, with S1 only connected with ipsi-lesional regions. This asymmetry correlates with neurological and motor impairment. Furthermore, when combined with lesion anatomical measures, S1 connectivity might be an important marker in explaining stroke outcome.

## Introduction

Despite recent advances in the diagnosis and treatment of cerebrovascular disease, stroke remains the leading cause of adult impairment worldwide^1^. Identifying a way to accurately determine post-stroke outcome is crucial to achieve the best results in rehabilitation. Neuroimaging techniques have been used to study anatomical and functional markers with respect to neurological and motor impairment. Results of anatomical imaging studies suggest that both lesion size and corticospinal tract lesion load (CST-LL, i.e., overlap of the lesion with the CST) correlate with post-stroke neurological^2^, upper-^3,4^ and lower-limb^5,6^ motor deficit.

Anatomical markers indicate the extent of damage caused by stroke but do not however, provide key information on the amount of cerebral reorganization that can take place in intact and peri-infarct tissue^7^ that may significantly impact behavioral outcome. In fact, it has been shown that regions distant to the infarct play an important role in post-stroke neuroplasticity and functional recovery^8^.

Functional markers such as resting-state functional magnetic resonance imaging (rs-fMRI) allow for the assessment of whole-brain functional connectivity (FC) at rest by measuring spontaneous modulation in blood oxygenation level-dependent (BOLD) signal without any task, providing a measure of temporal correlation of blood oxygenation fluctuations between brain regions^9^ as a surrogate marker of synchronized neuronal activity at rest. Although typically unilateral, stroke induces interhemispheric changes. Not only does it reduce activity in the affected hemisphere but causes, via transcallosal inhibitory fibers, a release of activity in the unaffected hemisphere (transcallosal disinhibition). In patients with stroke, this increased activity in the non-lesioned hemisphere has repeatedly been demonstrated^10,11^ and this lack of inhibition from the lesioned hemisphere likely interferes with recovery^10,12^. Consequently, studies in chronic stroke combining anatomical and rs-FC of interhemispheric connectivity found that patients with less interhemispheric M1 rs-FC and lower fractional anisotropy are more impaired^13^.

Measures of FC might therefore be good candidates to further define behavioral outcomes post-stroke, in addition to anatomical markers. Most studies have focused on connectivity of the motor cortex region^13–15^; however, changes in FC in other regions may provide additional information on cerebral reorganization. For instance, changes in somatosensory cortex activity^16–19^ and sensory networks^20^ are known to accompany motor learning therefore, integrity of sensory cortex connectivity might be an important marker of motor function post-stroke. Rs-FC of the sensorimotor cortex (M1 and S1 together) have shown similar results as the motor cortex alone, namely that interhemispheric asymmetry is reduced with chronicity and that it correlates with motor impairment^14,15^. It remains unclear however, what the contribution of the rs-FC of the primary sensory cortex (S1) alone to neurological or motor impairment really is.

The purpose of the present study is thus to identify stroke-induced changes in rs-FC of the primary sensory cortex (S1). In contrast to previous studies, we extended our analysis to identify inter and intra-hemispheric connectivity independently to both M1 and S1 regions on both affected and non-affected hemispheres. In the same participants, we also assessed lesion size and CST-LL to determine if changes in interhemispheric rs-FC could, added with anatomical markers, better describe neurological and/or motor impairment post-stroke.

## Results

### Rs-FC in healthy controls

**Figure 1:**
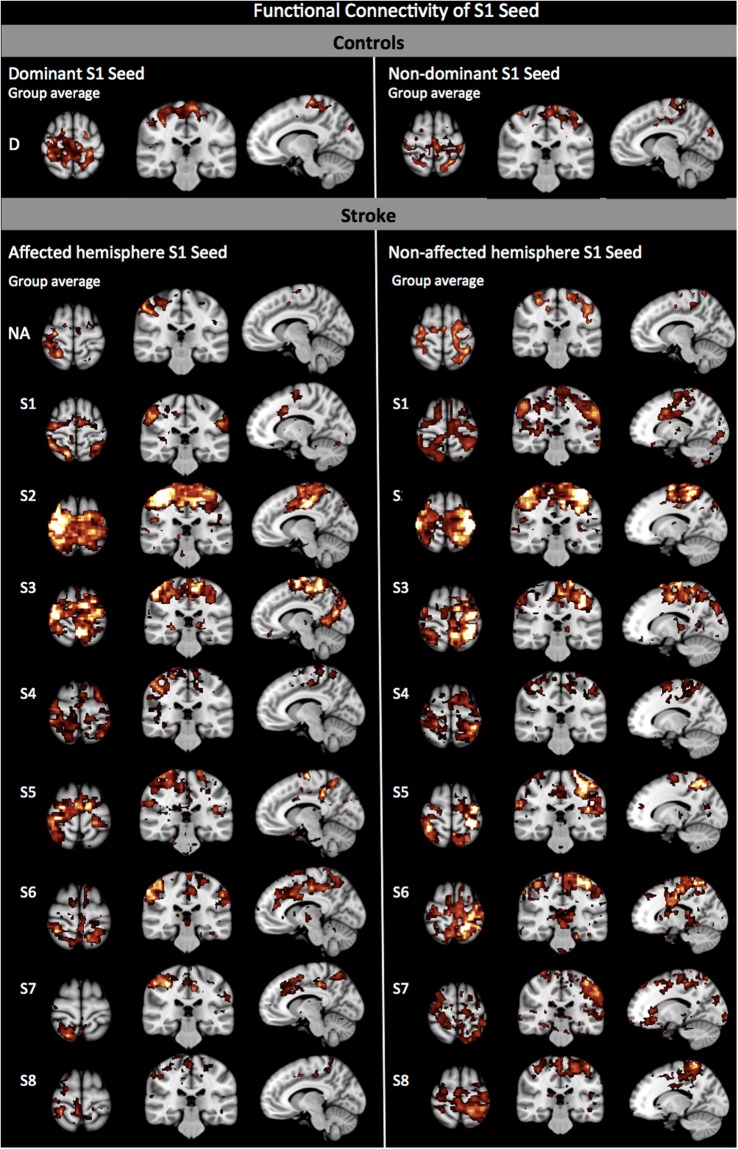
rs-FC in relation to S1-seed. Top panel shows control group and bottom panel shows post-stroke group connectivity maps when the seed is positioned over S1.

Positioning the seed on either dominant or non-dominant S1 showed both intra- and inter-hemispheric rs-FC between bilateral S1, occipital and premotor cortices (Fig. 1, top). In addition, parietal areas were also connected bilaterally, however, S1-parietal connectivity was mostly intra-hemispheric (BA5 and 7) when the seed was positioned over the non-dominant hemisphere. S1 connectivity was intra-hemispheric for the somatosensory areas (BA40 and 43) but also inter-hemispheric when the seed was positioned on the dominant side (BA40) only.

**Figure 2:**
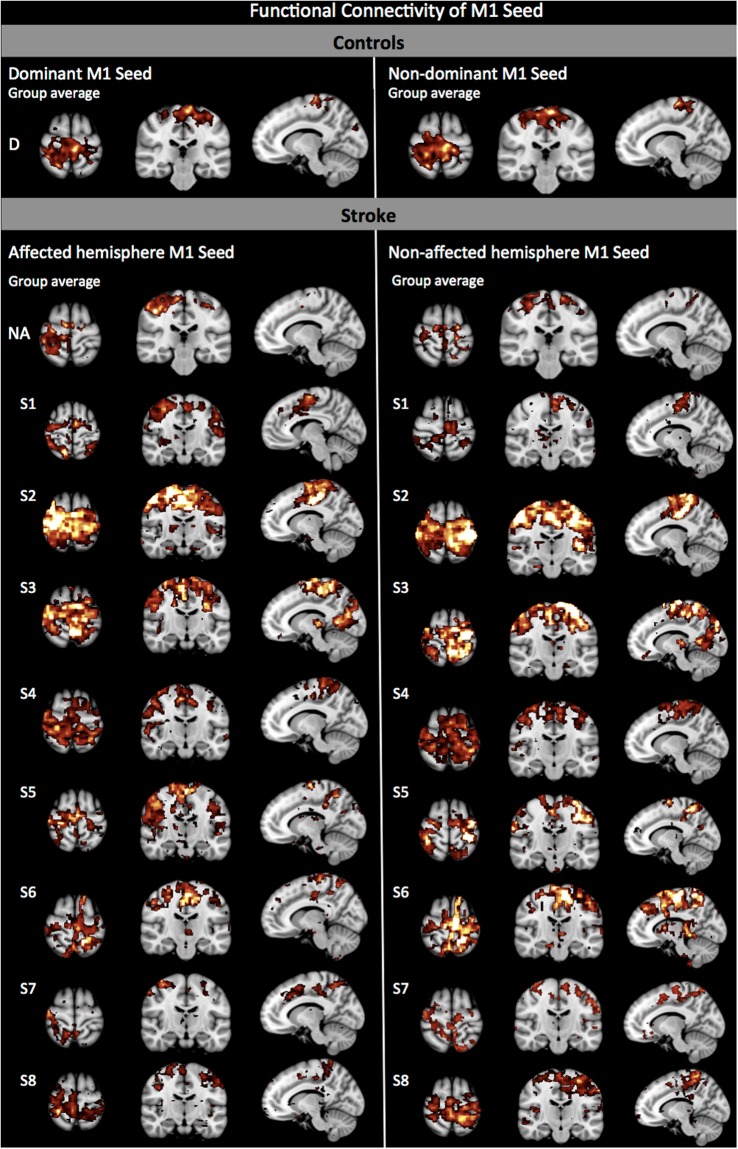
rs-FC in relation to M1-seed. Top panel shows control group and bottom panel shows post-stroke group connectivity maps when the seed is positioned over M1.

Positioning the seed over the dominant or non-dominant M1 in healthy controls revealed a symmetrical pattern of interhemispheric rs-FC in homotopic regions of the pre- and post-central gyri, parietal and occipital lobes (Table 1 and Fig. 2, top). Specifically, the seed was functionally connected to itself and to interhemispheric homologous region. There was intra-hemispheric connectivity of the M1-seed with ipsilateral parietal operculum in addition to inter- and intra-hemispheric connectivity observed with the sensory, parietal and occipital cortices. M1 intra-hemispheric, not inter-hemispheric connectivity was observed with the secondary somatosensory area (BA43).

**Table 1:** Clusters of significantly increased rs-FC in relation to each seed.

Seed location	Cluster Size (voxels)	Z	Max Voxels (mm)			Side	Broadmann Area (Topographical location of seed)
			X	Y	Z		
**Controls**							
D M1	11,789	5.31	−2	−30	68	ND D	3b*, 4a*, 4p*, 6*, 1, 2, 3a, 5L, 5M (M1: 3b*, 4a*, 4p*, 6*), 1, 2, 3a, 5L, 5M, **43**, **5Ci**
	1,037	4.22	−18	−82	48	D/ND	17,18
ND M1	11,754	5.42	−4	−28	68	D ND	3b*, 4a*, 4p*, 6*, 1, 2, 3a, 5L, 5M (M1: 3b*, 4a*, 4p*, 6*), 1, 2, 3a, 5L, 5M, **43**, **5Ci**, **7PC**
	615	4.05	16	−86	22	D/ND	18
D S1	10,340	5.2	−34	−38	56	ND D	1*, 2*, 3b*, 4a*, 5L, 6 (S1: 1*, 2*, 3b*, 4a*), 5L, 6, 7PC, 40, **3a**, **4p**, **5M**, **7A**, **43**
	1,455	4.42	−6	−84	28	D/ND	17, 18
ND S1	12,124	5.22	20	−36	68	D ND	2*, 3b*, 4a*, 3a, 4p, 5L, 5M, 6 (S1: 2*, 3b*, 4a*, 3a), 4p, 5L, 5M, 6, 1*, **7PC**, **40**, **43**
	1,100	4.24	−4	−84	28	D/ND	18
**Stroke**s							
A M1	8,246	5.46	44	−26	46	NA A	3b*, 4a*, 4p*, 6*, 2 (M1: 3b*, 4a*, 4p*, 6*), 2, **1**, **3a**, **5L**, **5M**, **5Ci**, **7PC**, **40**
NA M1	8,254	5.23	−48	−16	48	A NA	3b*, 4a*, 4p*, 6*, 1, 2, 3a, 5M (M1: 3b*, 4a*, 4p*, 6*), 1, 2, 3a, 5M, **5Ci**, **5l**, **7PC**
A S1	7,481	5.43	56	−24	52	NA A	2*, 3b*, 4p (S1: 2*, 3b*, 4p, 1*, 4a*), **3a**, **5L**, **6**, **7PC**, **40**
NA S1	7,320	5.12	−34	−44	64	A NA	4a*, 4p, 5Ci, 6, 40 (S1: 4a*, 1*, 2*, 3b*), 4p, 5Ci, 6, 40, **3a**, **5L**, **5M**, **7A**, **7PC**, **43**

R: Right; L: Left; S: subcortical; C: cortical; MCA: Middle Cerebral Artery; *Stroke size also represents the volume excluded for the resting-state analysis. ^* *^National Institute of Health Stroke Scale (0 = normal); ^†^Chedoke McMaster Stroke Assessment (7 = normal); *md:* missing data; participant could not be tested due to an acute injury suffered between image acquisition and clinical assessment.

### Rs-FC changes post-stroke

In subjects post-stroke, the pattern of interhemispheric rs-FC was asymmetrical. Using S1 as seed in stroke revealed the loss of most of the interhemispheric connectivity between the seed region and contra-lesional S1 (BA2 only; Fig. 1, bottom-left). The seed instead remained connected intra-hemispherically with itself. S1 connectivity with somatosensory areas was intra-hemispheric and only inter-hemispheric when the seed was on the non-affected S1. The S1-parietal connectivity was purely intra-hemispheric, in contrast with controls who did show inter-hemispheric connectivity between S1 and parietal areas. There was rs-FC between the S1 seed positioned over the affected or non-affected side with the premotor cortex, similar to controls however, the inter-hemispheric connectivity between affected S1 and contra-lesional premotor cortex was not present anymore. When the affected M1 was used as the seed region (Fig. 2, bottom-left), most of the sensory regions connected to the affected M1 were only intra-hemispheric (Table 1, Seed location A M1). M1 connectivity was only intra-hemispheric and on the affected side only (BA40). Furthermore, the interhemispheric M1-parietal connectivity observed in controls was not observed. Instead, M1-parietal connectivity was only present within the affected hemisphere (BA5 and 7). When the non-affected hemisphere was used as the seed region, a similar pattern was observed where connectivity between M1 (seed) and the parietal regions remained mainly within the non-affected hemisphere, where the seed was positioned. Connection of either S1 or M1 to the visual cortex was lost post-stroke.

### Anatomical and function markers in relation to stroke severity

Stroke-related neurological deficit, as measured with the National Institutes of Health Stroke Scale (NIHSS) score, strongly and significantly correlated with measures of laterality quantified by a laterality index (LI) of rs-FC with the affected S1 or M1 as the seed. It also correlated with the weighted CST-LL (*w*CST-LL) and lesion size, as shown in Table 2. Upper-limb impairment quantified by the Chedoke-McMaster arm and hand components also strongly and significantly correlated with LI of rs-FC with the affected S1 or M1 as seed, *w*CST-LL, and lesion size. For the lower-limb, only lesion size correlated strongly, but not significantly, with the Chedoke-McMaster foot and leg components. Weaker correlations were found for the foot component and rs-FC LI when the affected S1 or M1 were used as seeds, and wCST-LL. No correlations were found with the leg component.

**Table 2:** Anatomical and functional correlations with neurological deficit and motor impairment.

		NIHSS^b^	Chedoke-McMaster Stroke Assessment^c^			
			Upper Limb		Lower Limb	
			Hand	Arm	Foot	Leg
Anatomical	wCST	**0.764** ^**a**^	**−0.823** ^**a**^	**−0.768** ^**a**^	−0.459	0.072
	Lesion Size	**0.844** ^**a**^	**−0.804** ^**a**^	**−0.935** ^**a**^	**−0.803**	**0.616**
Functional	S1	**0.834** ^**a**^	**−0.784** ^**a**^	**−0.666** ^**a**^	−0.501	−0.153
	M1	**0.737** ^**a**^	**−0.697** ^**a**^	−0.524	−0.418	−0.023

Bold type indicates large correlation (r > 0.6 or <−0.6).

^a^Significant correlations, *p* < 0.05.

^b^To assess neurological deficit.

^c^To assess motor impairment.

To further evaluate the added value of rs-FC in determining post-stroke impairment, a hierarchical multiple regression analysis was performed. The lesion size, as the anatomical marker, was entered in the first block of the regression as it best correlated with NIHSS and all components of the Chedoke-McMaster than wCST-LL. For the second block, LI rs-FC with affected S1 as the seed was entered as the FC marker. LI rs-FC with affected S1 seed was selected for the model over LI rs-FC with M1 as both are highly and statistically correlated with each other (*r* = 0.852, *p* = 0.007) and with the NIHSS score. A very low level of multicollinearity was present (variance inflation factor <5 for all independent outcomes). Results of the hierarchical regression analysis with NIHSS as the dependent variable showed a significant regression equation with the lesion size explaining 66.1% of the variance (F = 11.69, *p* = 0.014). Adding the FC variable in the second block of the regression significantly improved the relationship (R^2^ change = 0.308 F = 79.028, *p* = 0.000), explaining 96.9% of the variance.

Results of the second hierarchical regression analysis with stroke impairment, as measured by the Chedoke-McMaster, showed that lesion size was a significant factor of arm (86%, *p* = 0.001), hand (60.3%, *p* = 0.023) and foot (68.5%, *p* = 0.022) impairment but not leg. The addition of LI rs-FC with the S1 seed in the second block increased arm (R^2^ change = 0.103, F = 76.55, p = 0.000) from 86% to 96.8% and hand impairment regression (R^2^ change = 0.268. F = 16.855, *p* = 0.006) from 60.3% to 87.1% and had no effect on the other components.

## Discussion

This study shows that after stroke there is a disruption of interhemispheric rs-FC between sensory areas. In contrast with previous studies that focused mainly on M1 alone or combined with S1 connectivity, we extended our analysis to identify inter and intrahemispheric connectivity to S1 and M1 regions separately. Our results suggest that after a stroke, there is a significant loss of S1 interhemispheric connectivity. Furthermore, S1 showed increased intra-hemispheric connectivity (mainly with premotor and parietal regions). Our results also demonstrate that after stroke a decreased connectivity between sensorimotor regions and the occipital cortex is present. Additionally, we showed that rs-FC between the primary sensory region may, when combined with anatomical measures of stroke lesion, be an important marker in describing stroke outcome. Moreover, lower-limb impairments are more difficult to describe in contrast to upper-limb impairment and overall neurological deficit.

The symmetrical interhemispheric rs-FC observed in control subjects resembles the sensorimotor network^21^ involving the precentral, postcentral gyrus and supplementary motor areas. As with other studies, we observed that the affected hemisphere S1 or M1 was mostly connected within that same hemisphere primary somatosensory regions^13,14,22^. Our findings support those that showed that a bilateral and symmetrical connectivity between hemispheres is crucial to achieve normal motor performance. In the current study, decreased rs-FC between ipsi-lesional S1 or M1 and contra-lesional sensorimotor areas and parietal region was demonstrated post-stroke. On the other hand, increased intra-hemispheric connectivity between sensorimotor areas and parietal cortex was observed. Instead, functional connectivity of either S1 or M1 with the occipital cortex was lost in stroke patients. These findings coincide with previous studies that reported changes in connectivity of the ipsi-lesional M1 with non-primary sensorimotor regions, such as the fronto-parietal cortex^23^, middle frontal gyrus, thalamus, cerebellum and non-motor brain areas such as the occipital cortex^22^. Our findings suggest that disruption of the symmetrical interhemispheric rs-FC may lead to changes in connectivity of ipsi-lesional brain regions to compensate for the loss of interhemispheric connection.

In contrast to previous studies, we additionally examined interhemispheric rs-FC when positioning the seed over the non-affected hemisphere S1 or M1 demonstrating a symmetrical and bilateral pattern of rs-FC. These results indicate that connectivity between homologous brain regions is not reciprocal but is rather lesion-dependent. This could be explained by the transcallosal disinhibition hypothesis, which proposes that after a stroke, cortical excitability is decreased due to the infarct in the affected hemisphere, therefore the affected hemisphere can no longer exerts its normal inhibitory influence on the non-affected hemisphere^10^, and/or the non-affected hemisphere exerts too much influence over the already affected one^24^. Thus, stroke can affect regions distant to the infarct compromising the functional network involved in sensorimotor performance and changes in inter and intra-hemispheric rs-FC of the sensorimotor network which might reflect changes in response to post-stroke damage.

Upper-limb motor performance has been correlated with interhemispheric rs-FC between sensorimotor regions as measured with the upper-extremity Fugl-Meyer^13,25^ assessment and Action Research Arm Test score^14^. In addition, *w*CST-LL and lesion size have also shown correlations with upper-limb motor impairment as assessed by the NIHSS arm motor score^3^ and UE-FM^4^. In our sample, both wCST-LL and lesion size were good markers of upper-limb impairment. Lesion size has been known to correlate with motor and neurological impairment^2^ while CST damage has been shown to contribute mostly to upper-limb impairment in patients with subcortical infarct^3^, but not in those with combined cortical and subcortical lesions^26^. Thus, the stronger correlations observed in our sample with lesion size is likely attributable to the presence of both upper and lower limb deficits as well as both cortical and subcortical infarcts. The addition of rs-FC when the seed was positioned over the affected S1 further decreased the variance of the model by 31% for the upper-limb impairment. However, the current analysis showed that neither anatomical markers (wCST-LL or lesion size) were good indicators of lower-limb impairment. Adding a functional marker did not improve the relationship. Recent studies have demonstrated that post-stroke patients were able to walk even after damage of the CST suggesting that lower-limb performance is dependent on other brainstem tracts such as the reticulospinal-tract^27^, vestibulospinal-tract, rubrospinal-tract and projections from the contralesional cortex^28^ which might play a major role in lower-limb impairment or spinal motor programs may play and additional role. Thus, lower-limb impairment should be considered differently than the upper-limb when evaluating potential for functional recovery. Findings in our study suggest that *w*CST-LL is a unique imaging surrogate marker only for upper-limb post-stroke motor outcome. Lower-limb impairments are more difficult to model –based on the integrity of a single tract– compared to upper-limb impairment and overall neurological deficit.

Despite its novel results, our relatively small number of subjects requires more investigation to generalize our results and to assess whether data from the acute state could predict outcome earlier in the recovery. The seeds and masks were generated using the Jülich atlas, therefore creating reproducible masks in MNI space that are 90% inclusive for both S1 and M1. Because these masks include neurons that cross the theoretical boundaries of a given area, there exists an unavoidable overlap between masks. Although we observe a difference between S1 and M1 connectivity, suggesting that the differences we observe are region specific, a residual uncertainty as to localization remains, which is inherent to cytoarchitectonic probability maps. Functional connectivity studies have been conducted in stroke yet, the changes in tissue composition at the site of the lesion is at various stages of necrosis and gliosis, affecting the BOLD signal^29^. Although we focused our analysis in regions remote to the infarct, it cannot be excluded that the change in tissue composition might have an effect on functional connectivity. In addition, it cannot be excluded that the difference in echo-planar imaging resolution might be an additional source of variability in our group comparisons. Adding longitudinal data would further help determine the predictive value of rs-FC markers for motor impairment.

Our results demonstrate that stroke can affect the functional connectivity of regions distant to the infarct, specifically S1, potentially further compromising motor performance. In addition, we showed that asymmetry in primary sensory rs-FC, when combined with anatomical measures of stroke lesion size, can be an important marker defining post-stroke outcome. Determining the amount of impairment and especially potential for recovery, as suggested by the amount of S1 symmetry, could potentially contribute to the optimization of clinical decisions and treatment post-stroke. This study highlights the important contribution of neuroimaging techniques may play to understand the brain’s reorganization after a stroke.

## Methods

### Subjects

Eight subjects (mean age: 67 SD 8 years; 5 males, Table 3) in the early chronic phase (>3 months) after a first ischemic cortical or subcortical stroke resulting in upper or lower-limb motor impairment (Chedoke-McMaster^30^ <7), and eight healthy age-matched subjects (mean age: 67 SD 9 years; 5 males) participated in this study. Healthy subjects were recruited from the community and had no history of other neurological, cardiovascular or psychiatric diseases. Handedness was assessed with the Edinburgh Handedness Inventory. All participants had scores >0.75 or <−0.75, hence no mixed dominance (1 control was left handed). Prior to their participation, all subjects gave their informed consent to this study that was approved by the McGill Faculty of Medicine Institutional Review Board regulations for human subjects’ studies. All methods were carried out in accordance with approved institutional guidelines and regulations.

**Table 3:** Stroke patient characteristics.

Subject	Sex	Age (years)	Stroke location	Stroke size* (mm^3^)	Lesion Load in CST (mm^3^)	Time Since Stroke (months)	NIHSS score^**^	Chedoke McMaster score^†^			
								Arm	Hand	Leg	Foot
S1	M	63	L, S, Subcortical	3548	868	3	1	6	6	6	5
S2	M	66	R, S, Subcortical	5412	68	10	1	6	6	5	5
S3	F	57	R, C, Central	5445	2209	3	1	6	6	6	7
S4	F	67	R, S, Posterior Limb Internal Capsule	325	265	6	2	7	7	6	*md*
S5	M	60	L, S, Basal Ganglia	4339	1550	20	4	5	3	6	5
S6	M	69	L, S, Basal Ganglia	13583	3350	23	6	3	2	6	4
S7	M	61	R, C, MCA	34274	1792	6	6	2	2	5	3
S8	M	57	R, S, MCA	28204	3611	72	7	2	2	5	2

R: Right; L: Left; S: subcortical; C: cortical; MCA: Middle Cerebral Artery; *Stroke size also represents the volume excluded for the resting-state analysis. ^* *^National Institute of Health Stroke Scale (0 = normal); ^†^Chedoke McMaster Stroke Assessment (7 = normal); *md:* missing data; participant could not be tested due to an acute injury suffered between image acquisition and clinical assessmen.

### MRI acquisition

Images were acquired on a Siemens 3T Trio Scanner at the Montreal Neurological Institute (MNI). Subjects were instructed to relax and keep their eyes open without focusing on any particular mental activity. T1-weighted anatomical images were acquired (TE = 2.98 ms; TR = 2300 ms, flip angle = 9°, 192 slices, 1 mm^3^ isotropic), followed by the acquisition of a 5-minute sequence BOLD echoplanar images (TE = 30 ms, TR = 2.3 s, 38 slices, 3.5 mm isotropic for S3, S4, S8 and all age-matched subjects and TE = 30 ms, TR = 1.81 s, 34 slices, 4 mm isotropic for S1, S2, S5 and S7). S6 was acquired on a 1.5T Siemens SonataVision Scanner (T1: TE = 9.2 ms; TR = 22 ms; flip angle = 30°, 176 slices, 1 mm^3^ isotropic and BOLD TE = 50 ms, TR = 2.03 s, 24 slices, 4 mm isotropic).

### MRI Analysis

#### Lesion

**Figure 3:**
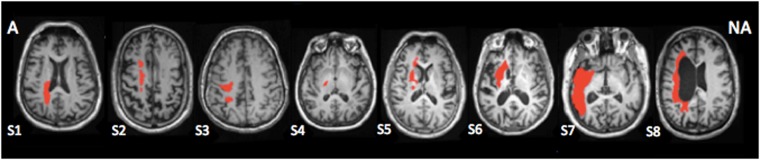
Lesion location. (**A**) Affected; NA: Non-affected. Location of the lesion superimposed in each individual T1-weighted image. The slice with the largest lesion size is presented.

Prior to the analysis, images of the left handed control and patients who had left hemisphere lesions were mirrored along the Z-plane so that all dominant hemispheres and all lesions would be on the same side for each group. Stroke lesions were manually outlined in each subject T1-weighted image (Fig. 3). Final infarct segmentation was independently reviewed by a stroke neurologist (AT) and segmentation was adjusted if necessary. To avoid the presence of artifacts due to the loss of neural activity at the tissue of the infarction, voxels identified as part of the stroke volume were excluded from the resting-state analysis.

#### Lesion Load

To assess damage to the WM fibers forming the CST, a lesion load (LL) of the affected hemisphere CST was calculated. The overlap volume of the lesion with a probabilistic CST from the Jülich atlas^31^ was computed according to the *w*CST-LL method^3^. The probabilistic CST was linearly transformed into each individual’s native MRI space. For each subject, a weighting factor was obtained from the probability of each voxel in each lesion slice intersecting with the probabilistic CST. Then, the *w*CST-LL was calculated according to the following equation (1).

1 $$w\mathrm{CST} \mbox{-} \mathrm{LL}={\rm{\Sigma }}{n}_{{\rm{\max }}}[\frac{1}{10}\cdot {\rm{I}}(n)\cdot ({\rm{voxel}}\,{\rm{volume}})\cdot {\rm{weighting}}\,{\rm{factor}}]$$

where *n*_max_ is the total number of intersecting voxels between the lesion volume and fiber tract and I(*n*) is the probability of the *n*^th^ voxel (as represented in the probabilistic tract).

### Resting-State image preprocessing

Data analysis was performed using a resting-state pipeline developed by the Center for Research on Brain, Language and Music (www.crblm.ca) relying on FSL 5.0.8 (FMRIB Software Library, Oxford, UK)^32^ and Matlab software (www.mathworks.com). Image preprocessing consisted of: (1) Discarding the first 5 volumes in each scan series to allow steady-state imaging, (2) slice-time correction (using Fourier-space time-series phase shifting), (3) brain extraction using FSL Brain Extraction Tool^33^, (4) motion correction, using a 6-parameter affine transformation built-in in FLIRT (FMRIB Linear Image Registration Tool)^33^, (5) global intensity normalization, (6) spatial smoothing with a Gaussian kernel of FWHM 6 mm (7) temporal high-pass filtering (Gaussian-weighted least-square straight-line fitting with σ = 100.0 s). Two transformations were performed: (1) from functional image to T1-weighted anatomical image (using a 7-Degrees of Freedom (DOF) transformation), (2) from MRI native to MNI space (using a 12-DOF linear affine transformation; voxel size 2 × 2 × 2 mm).

### Seed masks

**Figure 4:**
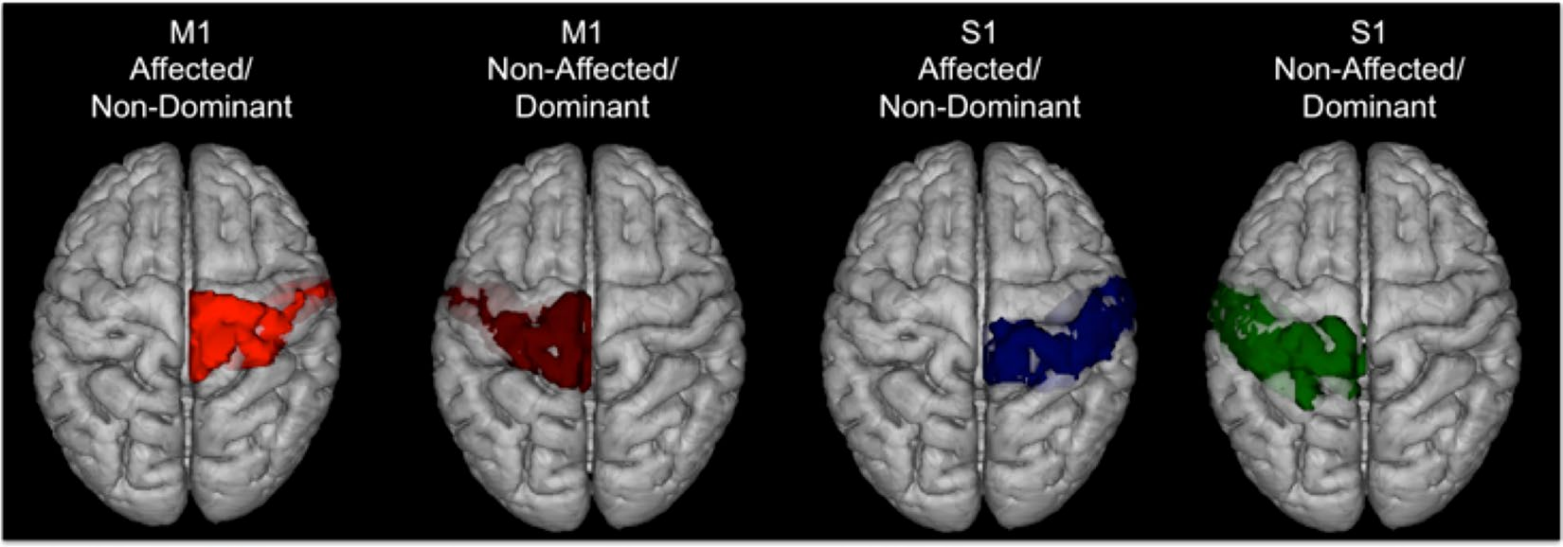
Schematic representation of the seed regions. Seeds were created using a combination of the probabilistic Jüelich atlas.

A seed-based connectivity analysis was used to identify voxels temporally correlated with BOLD signal fluctuations with bilateral M1 or S1. Masks were created in MNI space using a combination of the probabilistic Jüelich atlas^34^ BA4a and BA4p for M1 and BA1, BA2, BA3a and BA3b for S1 (Fig. 4). The Jülich atlas is a cytoarchitectonic probability map, which includes S1 or M1 neurons that lay outside the typical Brodmann area boundaries. The resulting masks were thresholded at a voxel probability of 10% (ensuring that most voxels associated with the ROI were included), binarized, and linearly transformed into MNI space. The resulting M1 seed thus included 99% of BA4a and BA4p on both right and left sides but also intersected with BA6 (36% left and 38% right) and BA3b (81% left and 60% right). The resulting S1 seed comprised 99% of BA3b on the left and 98% on the right, 96% of BA2 on the left and 93% right, 92% of BA1 on the left and 97% on the right, but also overlapped with BA4a (63% left and 66% right). We accepted this overlap with adjacent regions because we wanted to ensure that >90% of the target region was used as seed.

### Resting-state FC map computation

The BOLD signal from the preprocessing step was entered in a regression analysis, using a general linear model with the seed region time-series and the nuisance variables time-series as dependent variables, with FEAT (FMRIB Expert Analysis Tool)^35^. Physiological noise removal improves the results of the FC analysis at rest, therefore, the time-series for nuisance variables was calculated and excluded from the analysis as follows: first, the time series for Cerebrospinal Fluid (CSF), WM, and grey matter (GM) were extracted. The resulting segmented CSF, WM and GM images were thresholded (ensuring 80% tissue type probability) and transformed into functional space. The mean time-series was calculated by averaging the times-series from all voxels within the seed region. A total of nine nuisance regressors were used: CSF, WM, global signal, and six motion parameters (*x*, *y*, and *z* translations and rotations obtained from the motion-correction step). Relative motion was similar between groups (stroke: 0.06 SD 0.03 mm vs. controls 0.07 SD 0.05 mm, t = −0.457, p = 0.654). For each individual, a separate multiple regression analysis was performed on the time-series of the nuisance signal using FEAT. This way, the nuisance signals are removed and the residual image represents the temporal correlation of the corrected BOLD signal in the seed region with each voxels forming rs-FC maps with cluster thresholding at *Z* > 2.3, *p* = 0.05 corrected using a Gaussian Random Field (GRF) theory.

### Lateralization Index

To quantify changes in inter-hemispheric FC, a LI was calculated. All voxels functionally connected to M1 on each hemisphere were calculated as a mean difference ratio for the seed positioned over the affected M1. The same procedure was repeated for the seed position over the affected S1 but for the mean difference ratio of bilateral S1. The same masks used to generate the seed region were used to calculate LI: (ΣFC_A/D_ − ΣFC_NA/ND_)/(ΣFC_A/D_ + ΣFC_NA/ND_) where ΣFC_A/D_ denotes the number of voxels functionally connected to the seed inside the affected (patients) or dominant (controls) sensory or motor area (intrahemispheric connectivity) and ΣFC_NA/ND_ is the number of voxels functionally connected to the seed inside the non-affected/non-dominant hemisphere (interhemispheric connectivity). LI closer to 1 thus indicates connectivity within the affected (or dominant) hemisphere, i.e., more intra-hemispheric connectivity whereas an LI closer to −1 within the non-affected (or non-dominant), would indicate mainly inter-hemispheric connectivity (i.e., seed to opposite hemisphere, not to itself). Values close to 0 indicate symmetry, where intra- and inter-hemispheric connectivity is similar.

### Statistical Analyses

*Z*-statistic images from the individual-level analysis were entered into to a group-level analysis GLM using FEAT mixed-effects model with a cluster thresholding *Z* ≥ 3, *p* ≤ 0.05 corrected using a GRF theory. Significant areas connected to the seed and their locations in MNI coordinates were identified.

Pearson’s correlation was used to determine whether a subject’s anatomical and functional markers correlated with their NIHSS and Chedoke-McMaster upper and lower-limb scores. Hierarchical regression analyses were performed using *w*CST-LL, lesion size and LI values as independent variables to model post-stroke impairment.

### Data availability

The datasets generated during and/or analyzed during the current study are available from the corresponding author on reasonable request.
